# Experience-based medicine: a new paradigm

**DOI:** 10.1007/s00406-026-02321-y

**Published:** 2026-08-02

**Authors:** Stefan Leucht, Selina Hiller, Josef Priller, Kerem Böge, Markus Seidel, Alessandro Rodolico

**Affiliations:** 1 Department of Psychiatry and Psychotherapy, Technical University of Munich, TUM School of Medicine and Health, Klinikum rechts der Isar, Munich, Germany; 2 German Center for Mental Health (Partner Site Munich-Augsburg), Munich, Germany; 3 Neuropsychiatry and Laboratory of Molecular Psychiatry, Charité-Universitätsmedizin Berlin, Berlin, Germany; 4 University of Edinburgh and UK DRI, Edinburgh, UK; 5 Department of Psychiatry and Neurosciences, Charité Universitätsmedizin Berlin, Berlin, Germany; 6 Department of Psychology, Medical University Brandenburg, Neuruppin, Germany; 7 German Center for Mental Health, Partner Site Berlin, Potsdam, Germany; 8 Technical University of Munich, Circular Economy and Sustainability Assessment, Munich, Germany

Evidence-Based Medicine (EBM) is the combination of the best scientific evidence, patient preferences, and clinical experience; however, this final component has largely been neglected in the scientific literature. As EBM pioneer David Sackett wrote in 1996: “The practice of evidence based medicine means integrating individual clinical expertise with the available external clinical evidence from systematic research [1].” When the term EBM was first introduced in 1991 [2], the concept was heavily debated, amid fears that it would lead to “cookbook medicine” and eliminate the art of medicine. Systematic reviews and meta-analyses were also criticized for comparing apples and oranges. Nowadays, EBM is a universally accepted concept. Its first component—using scientific evidence for guideline recommendations—is firmly established, while the second—patient preferences and values—is expressed through shared decision-making [3, 4]. In contrast, the third component—clinical expertise—remains largely unaddressed. In this editorial, we present the website www.illuminatum.com, a first attempt to collect international expertise on treating severe mental illnesses and to outline how such a system can eventually integrate real-world experience into guidelines. The name is an allusion to enlightenment (Latin: lumen, luminis = light; illuminare = to enlighten), which aligns with the core principles of both Evidence-Based Medicine and the name of our university—Technical University of Munich (TUM).

An overview of the three EBM components.

*Evidence*: The randomized controlled trial (RCT) remains the cornerstone of scientific evidence. Increasingly, large-scale—ideally national—registry studies are being utilized to enhance generalizability. Today, the sheer volume of published RCTs requires them to be synthesized through systematic reviews and meta-analyses, the numbers of which have skyrocketed. Evidence synthesis methods have become highly refined; specialized approaches now include network meta-analyses, dose-response meta-analyses, individual patient data meta-analyses, component-level reviews, diagnostic-test meta-analyses, and systematic reviews evaluating the quality of rating scales using frameworks like COSMIN. Furthermore, sophisticated methods such as the PRISMA statement, Risk of Bias tools, and the GRADE approach are readily available to improve the scientific rigor of these reviews. Ultimately, systematic reviews inform clinical practice guidelines. As their methodological quality has continuously improved over the decades, the current gold standard has evolved into “living” guidelines—dynamic documents that are updated continuously and published electronically online.

The second component of evidence-based medicine—systematically including *patient preferences and values* in treatment decisions—is addressed through shared decision-making. In this process, patients are informed about the pros and cons of various treatment options so they can actively participate in a joint decision with their physician [4]. While significant progress has been made, the primary challenge remains how to present complex evidence so that laypeople can understand it quickly and use it effectively during consultations. Smartphone applications offer a natural delivery format, but in the field of psychiatry, they are still in their infancy.

However, almost nothing has been done so far to systematically implement the third pillar: *clinical expertise*. A PubMed search using our specific search string yielded only 303 hits, many of which were reports on guidelines partially based on expert experience. But this is not what we mean. By “experience-based medicine,” we instead mean utilizing the collective experience of the “crowd” of healthcare workers. How to leverage this collective experience is not well covered even in the “bible” of the field, the *Users’ Guides to the Medical Literature* [4]. One reason for this omission is that the founders of evidence-based medicine originally defined expertise as the experience of the individual doctor. While physicians have always relied on personal experience, their joint experience has never been systematically gathered and utilized. Our goal is to systematically collect and analyze the international expertise of physicians to ultimately integrate it into treatment guidelines. For example, if 500 clinicians state that they would proceed in a certain way in a specific clinical scenario, this practice-based knowledge could dynamically supplement evidence-based guidelines. Importantly, we do not mean traditional “expert consensus,” of which many already exist; rather, we aim to tap into the crowd intelligence of clinicians and frontline mental health workers. At a later stage, the personal insights of individuals with lived experience will be gathered as well. This experiential supplementation is particularly crucial in areas where scientific evidence is sparse or unavailable. While scientific evidence can define a broad clinical framework, the more specific a clinical question becomes, the less data is available—leaving ample room for expertise-based recommendations.

**Fig. 1:**
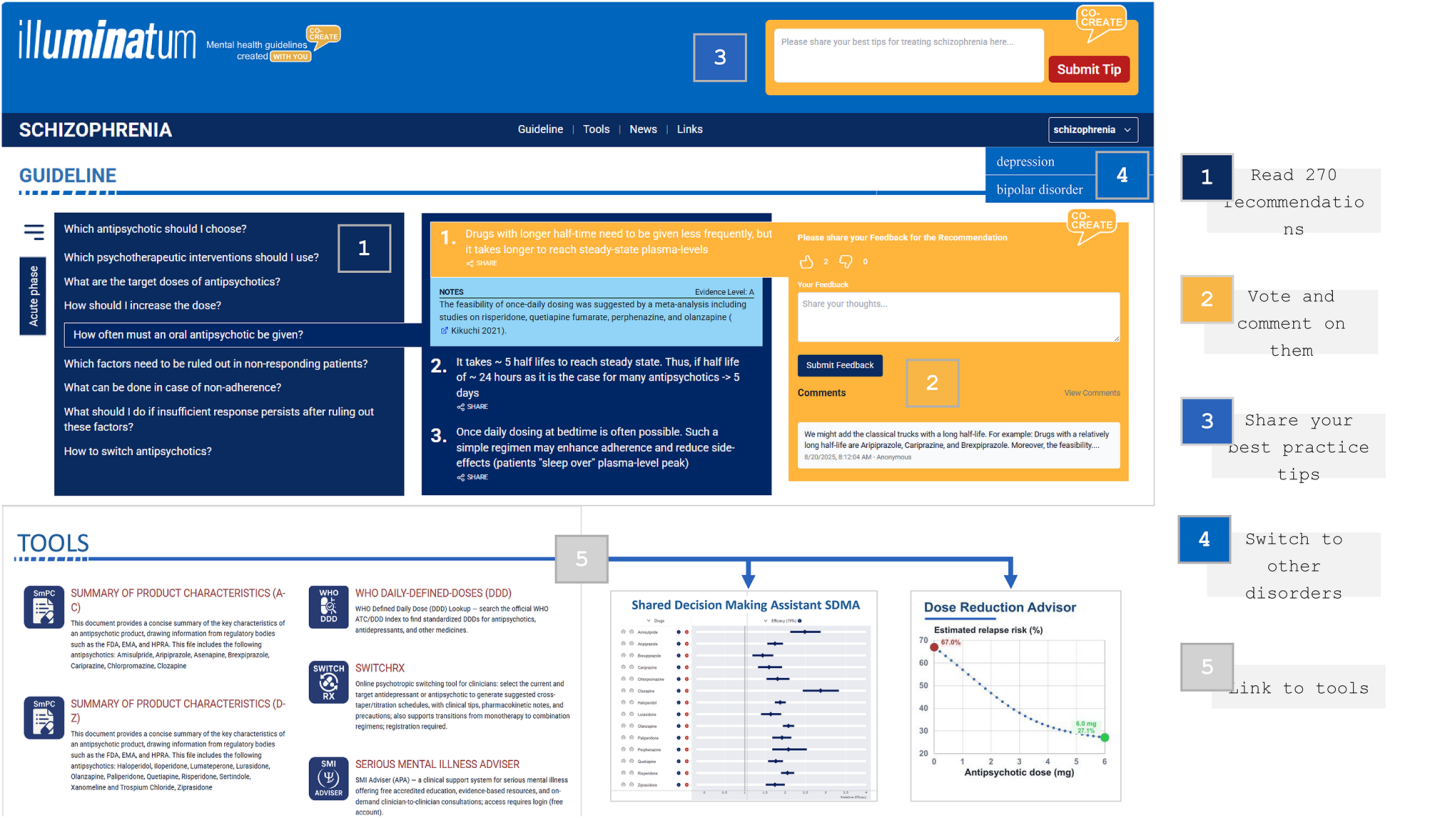
Explanation of the illuminatum.com website

The website www.illuminatum.com aims to integrate all three components of EBM. At its core are evidence-based living guidelines, currently featuring modules for schizophrenia spectrum disorders and major depressive disorder (see Fig. 1, point 4). We plan to develop guidelines for all major psychiatric disorders to ensure the platform serves as a comprehensive point-of-care resource for daily clinical practice. At the bottom of the site, users are directed to additional practical resources, including tools for selecting antipsychotic medications, a Shared Decision-Making Assistant [5], and aids for antipsychotic dose conversion, dose reduction, and switching strategies. The section also includes drug interaction checkers, summaries of product characteristics, links to external guidelines, and PowerPoint presentations for psychoeducation, among many others (see Fig. 1, point 5). Because these guidelines are “living,” they will be rapidly updated as soon as recommendation-altering evidence emerges. Our “evidence-to-decision” process relies primarily on the quality and strength of the scientific evidence; while factors such as availability, cost, or feasibility vary greatly by region, www.illuminatum.com remains international in scope. To maintain real-time updates, we run automated PubMed searches daily—one focusing on schizophrenia generally and another specifically tracking systematic reviews in the field. The guidelines comprise drug treatment and psychotherapeutic/psychosocial interventions. They are structured into distinct thematic blocks: acute treatment, special populations/subgroups, maintenance treatment, and side-effect management. Users can quickly access a specific recommendation (out of 270 total) alongside its scientific justification (see Fig. 1, point 1), with direct links to the primary source PDFs for interested readers. To foster collective expertise, the site features a forum where readers can vote, read and discuss the comments of colleagues, provide feedback, ask questions, and contribute their own real-world practice tips (see Fig. 1, point 2).

This forum is the primary vehicle for operationalizing experience-based medicine. Participants can vote on current recommendations using thumbs-up or thumbs-down indicators, allowing us to gauge consensus on the guidelines. Additionally, participants can make comments rooted in their real-world clinical experience treating schizophrenia rather than purely published evidence. At the top of the portal, users can share general “practice tips” that are independent of specific recommendations (see Fig. 1, point 3). We will analyze these experience-based responses using artificial intelligence (AI) tools to synthesize them into formal, experience-based recommendations. These insights will then be integrated back into the guidelines, initiating a new cycle of dynamic discussion and consensus-building. The principle is “co-creating” the guidelines together and leveraging the international experience.

This editorial serves as a catalyst for what we hope will be a wave of future projects dedicated to integrating clinical experience into guideline development. We are launching this initiative with a research project on schizophrenia. Those interested in the project are invited to visit our free platform at www.illuminatum.com, where further details regarding our study are available at the login page. www.illuminatum.com can also be used without participating in the study. We are looking forward to your participation.

**Pubmed search 29th May 2006**

( ( collective[tiab] AND ( intelligence[tiab] OR insight*[tiab] OR expertise[tiab] ) ) OR ( wisdom[tiab] AND ( crowd[tiab] OR crowds[tiab] ) ) OR ( crowdsourc*[tiab] AND ( physician*[tiab] OR clinician*[tiab] OR doctor*[tiab] OR expert*[tiab] ) ) ) AND ( ( treatment[tiab] AND recommendation*[tiab] ) OR ( clinical[tiab] AND recommendation*[tiab] ) OR ( clinical[tiab] AND management[tiab] ) OR ( patient[tiab] AND management[tiab] ) OR ( practice[tiab] AND guideline*[tiab] ) OR consult*[tiab] ) AND ( physician*[tiab] OR clinician*[tiab] OR doctor*[tiab] OR psychiatrist*[tiab] OR ( medical[tiab] AND expert*[tiab] ) OR ( clinical[tiab] AND expert*[tiab] ) )
